# Resilience in Web-Based Mental Health Communities: Building a Resilience Dictionary With Semiautomatic Text Analysis

**DOI:** 10.2196/39013

**Published:** 2022-09-22

**Authors:** Yong-Bin Kang, Anthony McCosker, Peter Kamstra, Jane Farmer

**Affiliations:** 1 Australian Research Council (ARC) Centre of Excellence for Automated Decision-Making and Society (ADM+S) Swinburne University of Technology Victoria Australia; 2 Social Innovation Research Institute Swinburne University of Technology Victoria Australia

**Keywords:** resilience dictionary, mental health, peer-support forum, topic modeling, text analysis, content moderation

## Abstract

**Background:**

Resilience is an accepted strengths-based concept that responds to change, adversity, and crises. This concept underpins both personal and community-based preventive approaches to mental health issues and shapes digital interventions. Online mental health peer-support forums have played a prominent role in enhancing resilience by providing accessible places for sharing lived experiences of mental issues and finding support. However, little research has been conducted on whether and how resilience is realized, hindering service providers’ ability to optimize resilience outcomes.

**Objective:**

This study aimed to create a resilience dictionary that reflects the characteristics and realization of resilience within online mental health peer-support forums. The findings can be used to guide further analysis and improve resilience outcomes in mental health forums through targeted moderation and management.

**Methods:**

A semiautomatic approach to creating a resilience dictionary was proposed using topic modeling and qualitative content analysis. We present a systematic 4-phase analysis pipeline that preprocesses raw forum posts, discovers core themes, conceptualizes resilience indicators, and generates a resilience dictionary. Our approach was applied to a mental health forum run by SANE (Schizophrenia: A National Emergency) Australia, with 70,179 forum posts between 2018 and 2020 by 2357 users being analyzed.

**Results:**

The resilience dictionary and taxonomy developed in this study, reveal how resilience indicators (ie, “social capital,” “belonging,” “learning,” “adaptive capacity,” and “self-efficacy”) are characterized by themes commonly discussed in the forums; each theme’s top 10 most relevant descriptive terms and their synonyms; and the relatedness of resilience, reflecting a taxonomy of indicators that are more comprehensive (or compound) and more likely to facilitate the realization of others. The study showed that the resilience indicators “learning,” “belonging,” and “social capital” were more commonly realized, and “belonging” and “learning” served as foundations for “social capital” and “adaptive capacity” across the 2-year study period.

**Conclusions:**

This study presents a resilience dictionary that improves our understanding of how aspects of resilience are realized in web-based mental health forums. The dictionary provides novel guidance on how to improve training to support and enhance automated systems for moderating mental health forum discussions.

## Introduction

### Background

*Mental health* is fundamental to our individual and collective capabilities of thinking and interacting with each other and enjoying life [1]. Mental health challenges have been increasing worldwide, with consequent negative impacts on social and economic prosperity [2-4]. A 2017 survey [5] found that approximately 1 in 7 people (equivalent to 1 billion people) worldwide have experienced mental illness. In Australia, government statistical reports show that approximately 20% of people (4.8 million) experience mental health issues such as anxiety-related conditions and depression [6]. From 2020 to 2021 during the COVID-19 pandemic, 3.4 million Australians aged 16 to 85 years (17% of that age group) sought help from a mental health professional [7]. With its lockdowns and social distancing requirements, the COVID-19 pandemic is associated with what some have called an “unprecedented mental health crisis” [8], accelerating the need for community and digital interventions [9].

Reaching out to others for support is a vital part of enhancing resilience [10]. On this basis, it has been shown that *online peer-support mental health forums* (eg, those run in Australia by the charities Beyond Blue [11], SANE [Schizophrenia: A National Emergency] Australia [12], and ReachOut [13]) play increasingly important roles in establishing social connections, sharing knowledge and experiences, and providing emotional support among people with lived experiences of mental illness [4,14]. Such forums complement publicly funded and private health services by enabling firsthand access to people with shared experiences, advice, and guidance. Within forums, specially trained staff, volunteers, and untrained peers provide a supportive and safe space to talk and be heard [15,16]. Evidence shows that the benefits include the following [17]: (1) building safe and trusting relationships, (2) ensuring values of mutuality and reciprocity, (3) promoting validation and application of experiential knowledge, (4) enabling peers to exercise leadership through peer support, and (5) empowering peers to discover and make use of their own strengths. As complements to clinical and telehealth services, there remains a need to better understand the strengths of peer-support forums and optimize their management and moderation.

*Resilience* is an accepted strengths-based concept rather than a deficit or harms-based concept. Generating resilience is a regularly suggested approach to addressing mental health issues in community settings and falls within a preventive model of health care [8,18,19]. Resilience is understood variously across several disciplines as an individual and collective capability to adapt in the face of adversity, trauma, tragedy, threats, or even significant sources of stress [20]. In response to challenges, resilient individuals and communities can draw on psychosocial resources—personal and collective—to cope and adapt [4]. As a result, an individual’s resilience can positively affect their mental health. However, more research is needed to understand the characteristics of resilience and how it is realized through interactions within dedicated web-based mental health support settings, such as peer-support forums. This research contributes a method, as well as findings, that can help forum service providers demonstrate their outcomes and tailor their moderation practices to optimize resilience-building interactions.

### Prior Work

Despite the potential utility of online mental health peer-support forums for building resilience, *whether* and *how* *resilience is realized* by participants has received little research attention. Where resilience or strengths-based resources are the focus of existing studies, approaches fall into two streams: (1) qualitative analysis using user surveys and (2) qualitative and quantitative analysis of web-based forum data using either manual or larger-scale artificial intelligence techniques. The first stream is centered on manually identifying aspects of resilience and their reciprocal associations with mental health from web-based user surveys during particular events (eg, the COVID-19 pandemic [9,19]) or from certain groups (eg, university students [18] or adolescents [21]). User survey data are less adept at showing how resilience is realized through forum interaction and discourse as it is cross-sectional at points in time and asks specific questions created by survey designers, which might not resonate with forum users’ perceptions and experiences.

The second research stream has evolved with recent advances in natural language processing (NLP) and machine learning. These methods have shown promise as a way of using forum content as data sets for exploring public health questions [22]. However, much of this work has been “risk” and “harms” focused rather than strengths focused. For example, a triaging system was used to assist web-based peer support by classifying forum messages into different risk levels based on how urgently moderators’ attention was needed [23]. A study [3] compared 2 web-based depression forums (Beyond Blue [11] and *r/depression* on Reddit [24]) using NLP techniques based on user sentiments and discussion topics. NLP techniques have also been used to identify which posts from web-based health forums (HealthBoards [25], Inspire [26], and HealthUnlocked [27]) are related to the COVID-19 pandemic based on conversations among mental health consumers. Other studies have applied NLP to detect sentiments and emotions (ie, fear, anger, sadness, and joy) [28-30] for depression diagnosis [31,32] and to understand grief processes [33] and stress [34]. As noted, these studies tend to target risks associated with mental ill health, leaving a gap in identifying strengths-based interactions in web-based data sets and how web-based services might help generate positive health outcomes.

### Aims of This Study

As mental health services seek to optimize and expand digital support interventions, new methods of analysis and monitoring of forum activities are needed. In this study, we harnessed NLP techniques to reveal indicators of resilience and how resilience is realized within online peer-support forums. This work provides new insights into how resilience is realized in forums by building a *resilience dictionary* that reveals the resilience themes discussed across 2 years of forum activity.

More specifically, the resilience dictionary we generated shows (1) the types of themes that characterize resilience and (2) the relationships between different resilience indicators with respect to their realization. These insights reveal the broader (more abstract or common) and narrower (more specific) nature of specific resilience indicators. The resilience dictionary can be used by forum service providers and researchers to show that resilience is enabled by forums and how this occurs. It can help monitor changes in resilience realized by forum users over time and understand what prompts resilience changes, both within the forum and stimulated by external events. By establishing a method for quantifying resilience in unstructured text data, the resilience dictionary can help forum managers and creators to think more strategically about how to design the forums and maintain and moderate them more effectively. Revealing the characteristics of resilience and the extent to which it is realized over time can also help nonprofit organizations attract funding to sustain and further develop these forums.

To achieve our aims, we investigated the following research questions (RQs):

- RQ1: How can we characterize resilience indicators using topics (ie, major themes of posts) and their descriptive terms?
- RQ2: How are resilience indicators realized over time, and which are more dominant than the others?
- RQ3: How can we create a resilience dictionary that reflects the characteristics of each resilience indicator and the relatedness between the resilience indicators?

To address these RQs, we conducted a semiautomatic approach using methods that incorporate *topic modeling* (a type of statistical modeling in NLP) and exploit *human knowledge*.

To address RQ1, we explored optimal methods for discovering major thematic concepts, or *topics*, in forum data using topic modeling. Subsequently, using qualitative methods, we mapped these topics to the resilience indicators. The topics and their descriptive terms formed the controlled vocabulary of the resilience dictionary.

To investigate RQ2, we observed the *resilience prevalence* by analyzing the proportion of input forum data during the specified periods. We then analyzed the prevalence of each resilience indicator over time.

To explore RQ3, we created a resilience dictionary using the results of topics mapped to each resilience indicator. To identify the relatedness between resilience indicators in terms of their realization, we examined the co-occurrence patterns of resilience indicators from the input forums to automatically build a taxonomy of resilience indicators (ie, *resilience taxonomy*). This resilience taxonomy sheds light on whether resilience indicators are dependent on or independent of other resilience indicators.

For this work, we used data produced by SANE Australia’s 2 forums. SANE forums represent one of the largest web-based mental health peer-support communities in Australia, where people aged ≥18 years with mental health issues can register and engage in support, training, and education. Users can interact with other people (ie, peers) experiencing similar mental health challenges through the forums.

To our knowledge, this study is the first to explore the construction of a resilience dictionary by examining relationships between topics and resilience indicators and analyzing the relatedness between resilience indicators from web-based mental health forums using NLP and human knowledge. The significance of this study lies in the following aspects. First, we present how strengths-based resilience develops in web-based mental health forums based on a wider spectrum of web-based mental health discussions. Prior studies have attempted to accomplish this; however, they were small in scale and limited by specific events (eg, the COVID-19 pandemic) [8,9,14] or by certain age groups [2,18,21]. Second, the resilience dictionary can provide evidence-based information that can help design and maintain better mental health support services, as well as improve responses to people seeking mental health support. In particular, this benefit can be valuable for nonprofit mental health organizations that often have difficulty securing and allocating resources to improve their services. Finally, this work shows how mental health services can be made more effective by using support forum data, the value of which is becoming increasingly recognized [22,30].

## Methods

### Overall Design

#### Overview

**Figure 1:**
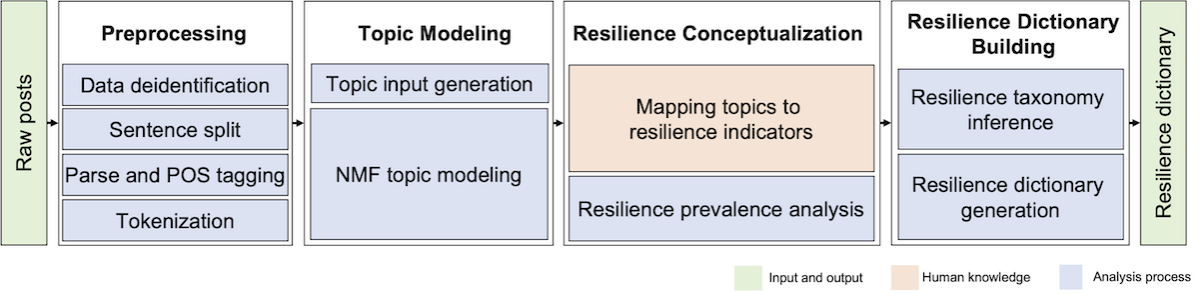
The 4-phase analysis pipeline that preprocesses forum posts, finds topics, conceptualizes resilience indicators, and builds a resilience dictionary. NMF: nonnegative matrix factorization; POS: parts of speech.

The 4 phases of our semiautomatic approach to creating a resilience dictionary are shown in Figure 1. Forum posts were the *first* preprocess to ensure deidentification and perform tokenization to generate meaningful terms. *Second*, topic modeling was used to discover the key topics (ie, themes) discussed in the posts. *Third*, the topics were mapped to a framework of resilience indicators using thematic coding processes. *Fourth*, a resilience taxonomy was established based on the co-occurrence analysis of resilience indicators from the posts. A resilience dictionary was then generated by integrating the resilience taxonomy with all the outcomes of the previous 4 phases. In the following sections, we present a detailed description of the phases after introducing the data set and the nature of the resilience indicators used in the study.

#### SANE Forum Data

SANE Australia is a national mental health charity that supports Australians affected by mental illness by providing peer-support services, training, and counseling. SANE data were collected from two web-based forums: (1) the “Lived Experience Forum” and (2) the “Friend, Family and Carers Forum.” The former is for people who live with complex mental health issues, whereas the latter is for their supporters and carers. SANE forums are moderated by mental health practitioners and aim to offer a safe and anonymous space on the web for discussing mental health and related issues [35]. Forums provide participants with social connections, allow the sharing of feelings and emotions, and provide a source of information generated through conversations. Forum discussions are intended to help consumers affected by mental illness explore positive pathways to address issues affecting their lives. According to the SANE annual report for 2020, there were 21,041 registered forum members, and 151,137 individual Australians used the forums in 2020 [35].

We obtained a national sample of posts from the SANE forums from 2018 Q3 (starting July 2018) to 2020 Q4 (ending December 2020). In total, 70,179 posts by 2357 users were obtained and used in this study in collaboration with the SANE forum managers.

#### Resilience Indicators

Drawing on the resilience framework proposed by Berkes and Ross [36], which combines psychological and community development approaches, we identified common components of personal and community resilience. Berkes and Ross [36] devised a conceptual framework of resilience pertaining to people’s lives in communities, allowing the concept to account for the wider ecosystem of factors affecting an individual’s mental health and well-being. Although there is a range of approaches to resilience, this framework is useful for understanding how resilience is realized by members of a forum, given their location in both physical on-the-ground communities and the web-based peer-based community of the forums.

The adopted resilience approach is premised on the understanding that people and communities that are resilient require access to a set of personal, interpersonal, or community resources. On the basis of research evidence, these involve *learning* or access to new knowledge, information, and skills, leading to an increased capability of dealing with change [37]; *social capital* or access to networks of people and the support, trust, and integration they can foster; a sense of *belonging*, including belonging to groups and places, acceptance as part of a group, and the processes that lead to identity formation [38]; *adaptive capacity* or using resources to enable adaptation and positive behavior change [39]; and *self-efficacy* or being able to self-organize or work toward feeling in a controlled state [36]. The definitions of these indicators and how they are conceptually realized in the context of mental health are shown in Table 1. In this study, the generation of the resilience dictionary was focused on and built from these 5 indicators.

Theoretically, we expect to see some variation in the way each indicator of resilience manifests in the web-based discussion context (in comparison with other contexts) and differences in their prevalence [36]. This means that some indicators should act as generators or resources, and others act as attributes or characteristics of resilience capacity, such as adaptive capacity or instances of self-efficacy. These dependencies were tested through RQ3, with the application of the taxonomic analysis described in phase 4.

**Table 1:** Resilience indicators, their definitions, and their conceptual realization in web-based forums.

Resilience indicators	Definition	Realization in community forums
Social capital [38]	Access to social networks and the support, trust, and social integration they can foster	Formation of social connections that individuals could draw on for confidence and support Expressing a willingness to share stories of similar experiences with mental health issues
Belonging [38]	Belonging to people and places, acceptance as part of a group, and the processes that lead to identifying formation	Introducing themselves to others Sharing stories and lived experiences with those experiencing similar challenges and participating at their own pace
Learning [37]	Access to knowledge, information, and skill development	Sharing applied and experiential knowledge with others Offering advice on how to practice good mental health and strategies on how to navigate mental health services
Adaptive capacity [39]	Use of resources that enable adaptation and positive behavior change	Self-reporting their own activity or behavior changes when responding to advice or information on the forum
Self-efficacy [36]	Ability to self-organize or work toward feeling in a controlled state	Communicating feelings of control in the aspects of their life or condition Supporting others by keeping them accountable for their changed behaviors or positive lifestyle choices that made them feel in control

#### Ethics Approval

The university ethics committee approved this study (R/2019/033). In addition, we adhered to SANE’s ethics and data governance policies throughout, which included establishing a data-sharing agreement, anonymizing posts to protect forum users’ identities, and applying a data security protocol.

### Phase 1: Preprocessing of Forum Posts

In the preprocessing phase, the goal was to break up each post into smaller basic units of meaning. User identifiers (IDs or names) were removed to deidentify the posts. Posts were then split into sentences to perform part-of-speech tagging and remove stop words (words such as *is*, *are*, and *the*, which do not carry useful information). The remaining terms were converted into their lemmatized forms to group the inflected forms of words together. Posts that were too short to add value to topic modeling (<5 terms) were removed. Finally, the remaining terms were used as the input to the next “topic modelling” phase.

### Phase 2: Topic Modeling

#### Overview

Topic modeling has gained attention because of its capacity to discover latent thematic topics in a large corpus of text documents [40,41]. A topic is a specific, recognizable theme defined by a cohesive set of terms representing the characteristics of that theme. Nonnegative matrix factorization (NMF) [42] was chosen as a method because of its effectiveness in discovering topics in short text documents and its ability to discover both broad and specific topics. As a method of analyzing forum posts, NMF can help identify both broad themes discussed often over time and more specific themes that might relate to periodic events [43].

A key idea of NMF is to decompose a term-document matrix *n* by *m* A (*n* is the number of forum posts, and *m* is the number of terms) into 2 nonnegative submatrices, *n* by *k* W (*k* is the number of topics) and *k* by *m* H, such that A is approximated by the multiplication of W and H, denoted as A **≈** W × H. In A, the weight of a term in a document is measured by a well-defined method, the term frequency–inverse document frequency weighting scheme [44], via term frequency and rarity. The matrix W represents the document membership weights over the 𝑘 topics. Each row denotes a document, and each column corresponds to a topic. Sorting the values of a column (topic) provides the ranking of the most relevant documents for the column. The matrix H contains the term weights relative to each of the 𝑘 topics. A row defines a topic. Sorting the values on each row provides the ranking of the most relevant terms (descriptors) of each topic.

#### Topic Input Generation

After preprocessing, each post was represented by a list of terms. Before applying topic modeling, we performed 2 steps to generate topic inputs. In the first step, the posts were split into disjoint collections based on the time duration of a “quarter” of a year. This collection period was the best fit for identifying granular topics (ie, neither too broad nor too specific). This generated 10 post collections from 2018 Q3 to 2020 Q4. The second step involved building a term-document matrix A using the term frequency–inverse document frequency weighting scheme from each collection.

#### Two-Layered NMF Topic Modeling

**Figure 2:**
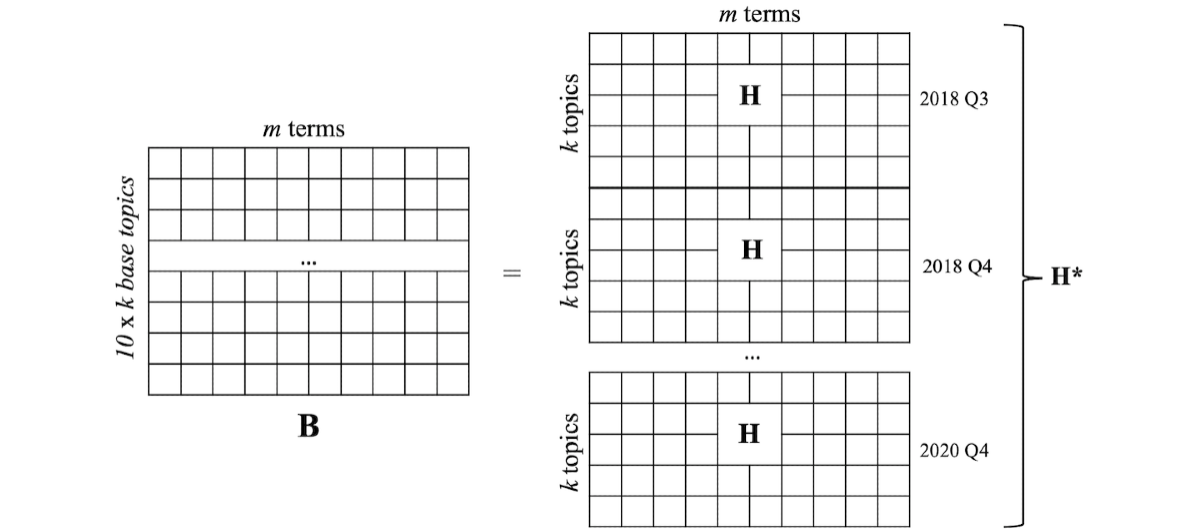
Conceptual illustration of constructing the matrix B by stacking up the matrices of H* generated by the first-layer nonnegative matrix factorization.

The 2-layered NMF approach [45] was applied to term-document matrices (denoted by A*) to discover both broader (longer-lived) and more specific (shorter-lived) topics. This process extracts broader topics observed across the entire time frame (2018 Q3 to 2020 Q4), as well as specific topics in each quarterly period, generating 2 topic layers. In the first layer, we find *k* topics, called *base topics*, from each term-document matrix A of A* using NMF, where *k* is a user-specified parameter. The output of this layer is a set of 2 matrices, W and H, from all disjoint post collections, denoted by W* and H*, respectively. In the second layer, we identified another type of topic, called *ensemble topics*, by analyzing similarities and variations among all the base topics generated from 2018 Q3 to 2020 Q4. A base topic-term matrix B was created by stacking each matrix H from H* (Figure 2). Each H comprises *k* topics with their constituent terms. In B, each row corresponds to a base topic, and each column is a term from the original posts. Each entry in B shows the weight (or importance) of the association of a term with a base topic, where the weight was inferred by the first-layer NMF. As shown in Figure 2, the dimension of B is 10 × *k* by *m*, as H*, which comprises 10 different matrices of H (from 2018 Q3 to 2020 Q4) and *m* terms.

From B, we applied NMF to produce *k′* ensemble topics, where *k′* is a user-specified parameter, thus producing B ≈ W' × H', where (1) W' denotes the base topic membership weights over the 𝑘′ ensemble topics, and (2) the H' matrix shows the weights of terms for each ensemble topic. To produce the membership weights of the original posts over an ensemble topic, we calculated the following expression:

D ≈ C × W'^T^ (equation 1)

Here, C is the document-base topic matrix made by stacking each matrix W of W* in the same way as B was created from the matrices of H*. D is the document-ensemble topic matrix and W'^T^ is the transpose of W'. Each row in D is an original post, and each column represents an ensemble topic.

### Phase 3: Resilience Conceptualization

#### Overview

In this section, we present how to conceptualize each resilience indicator using its relevant topics. In this context, conceptualization refers to the process of specifying the characteristics of resilience indicators based on their relevant discussion themes (ie, topics) from qualitative forum posts. First, we present how to characterize each indicator using the discovered topics. Second, we present a method to determine whether resilience indicators are time varying. This can provide additional insights into their dynamic characteristics over time.

#### Mapping Topics to Resilience Indicators

Here, our goal was to connect topics with relevant resilience indicators (ie, in relation to our predetermined resilience framework discussed previously). For this, we conducted qualitative content analysis, where knowledge of what happens on the forum and its aims is used to assess the meanings of topics and link each topic to relevant indicators. As qualitative data, we analyzed the meaning of the top *N* (*N* is a user-specified parameter) descriptive terms for each topic. As complementary data, we also examined the posts most relevant to each topic to analyze the meaning of the topic more rigorously in relation to the indicators of resilience. This content analysis was deductively performed using the framework of predefined resilience indicators. Note that a topic can be mapped to >1 resilience indicator depending on the thematic analysis. We followed a recent work showing the benefits of combining topic modeling with qualitative methods, particularly in the interpretation and contextualization phases of the analysis [46]. Here, our focus was on gaining an understanding of the nature of topics and their associations with resilience indicators based on domain knowledge of the context and posts that contain them most frequently. Topics cannot be meaningfully interpreted based only on the top *N* words as topics are not independent of the posts in which they appear.

The two types of qualitative data generated and analyzed were as follows.

First, the top *N* terms that best describe each ensemble topic (henceforth simply, “topic”) were identified from the topic-term matrix H' generated from the matrix B (recall B ≈ W' × H' presented in phase 2; Figure 2). Note that each row in H' indicates the weights of all terms with respect to a topic. We generated these top *N* terms by ranking the weights. Examining the collective meaning of these terms informed the mapping process of each topic to the relevant resilience indicators. For the value of *N*, we used 15.

Second, we associated each topic with its most relevant original topic. This association was identified from matrix D (equation 1), which signifies the membership weights of the original posts for the discovered topics. By ranking these weights, we identified the top *M* most relevant posts for each topic. In our study, we chose to use 20 as the value of *M*. By conducting a content analysis of these posts, we gained insights into the range of words and ideas surrounding each topic.

#### Resilience Prevalence Analysis

**Figure 3:**
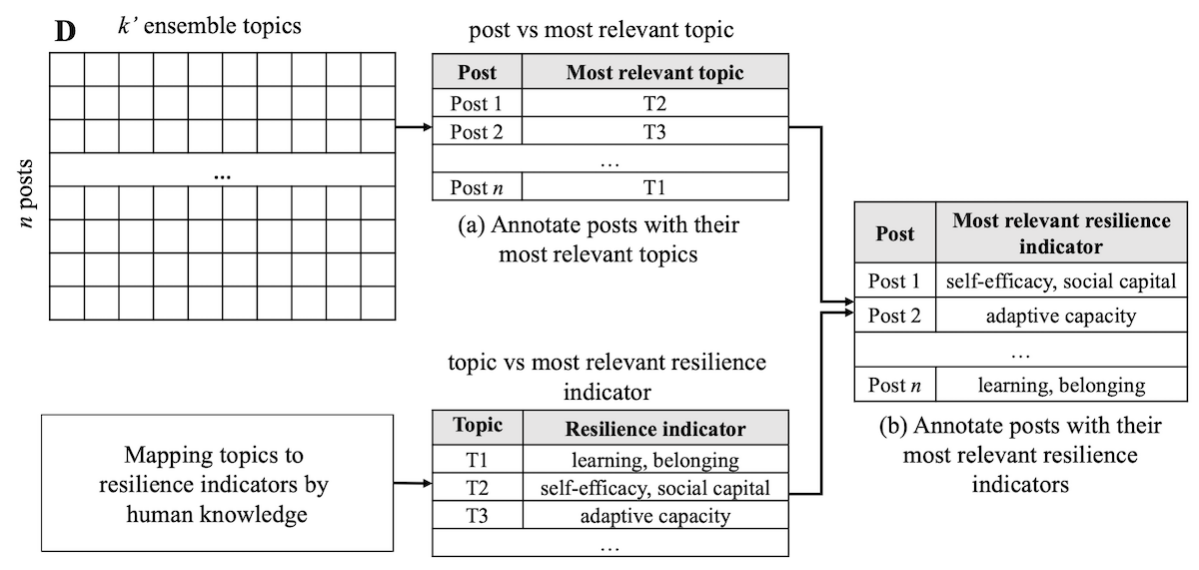
Illustration of the 2 steps used to annotate posts with their most relevant resilience indicators. The first step shows how to annotate each post with its most relevant topic from the document-ensemble topic matrix D in equation 1. The second step annotates each post with its most resilience indicators from the outcome of the first step and the mapped outcomes from topics to their most relevant resilience.

*Resilience prevalence* analysis can identify the dynamics of resilience indicators in terms of their realization over time. We estimated resilience prevalence as the proportion of the original posts associated with their most relevant resilience indicators. Using this analysis, we determined which resilience indicators were significantly or vaguely realized at a particular period. Thus, a resilience realization pattern over time was estimated using resilience prevalence analysis. To analyze resilience prevalence, it is essential to *annotate* each post with its most relevant resilience indicator(s). This was achieved by the following 2 steps. First, recall that from the document-ensemble topic matrix D (equation 1), we identified the membership weight of each post over each topic. On the basis of the weights of each post over all topics, we annotated each post with the topic that had the highest weight; thus, this topic was seen as the most significant topic for the post. Second, we note that each topic is mapped to its relevant resilience indicators in the previous step (see the *Mapping Topics to Resilience Indicators* section). Figure 3 illustrates these 2 steps using artificial examples.

### Phase 4: Resilience Dictionary Building

In this section, we present our motivation and approach for building a resilience taxonomy that is part of our resilience dictionary. We then elaborate on the definition and creation of the resilience dictionary.

#### Resilience Taxonomy Inference

Our objective in creating a resilience taxonomy was to improve our understanding of (1) the relatedness between resilience indicators in terms of their realization (ie, whether the indicators are independently realized or corealized) and (2) which indicators are likely to facilitate the realization of some other indicators. To achieve this objective, our approach exploits the realization co-occurrences of resilience indicators. These co-occurrences are observed through annotated posts (see the method in phase 3). The taxonomy can conceptualize the relationships of indicators in an automated way that informs the understanding of resilience realization relationships. In general, the key relationship in a taxonomy is the “is-a” relationship [47]. The “is-a” relationship has a hierarchical structure, and thus, its transitivity is logically inferred by navigating the hierarchical relationships between entities in a taxonomy. Intuitively, within our context, the higher a resilience indicator is positioned in the resilience taxonomy, the broader or more abstract it is. A lower position indicates greater specificity. Given a hierarchical path in the taxonomy between 2 indicators, we can derive the lower-ranked indicators that most likely influence the realization of higher indicator(s). As a result, semantic knowledge derived from the taxonomy can facilitate the corealization analysis of resilience indicators.

To create a resilience taxonomy, our fundamental goal was to examine the co-occurrences of resilience indicators annotated in the original posts. In particular, we used the popular subsumption method [48,49] that has proven to be effective for taxonomy learning in NLP; that is, from such co-occurrence knowledge, we build that a resilience indicator *x* subsumes another resilience indicator *y* if the posts annotated with *y* are a subset of the posts annotated with *x*. On the basis of this scheme, we can find taxonomic relations between resilience indicators.

#### Resilience Dictionary Generation

By aggregating the findings to this point, we generated a resilience dictionary that represents (1) how resilience indicators are characterized by particular topics, (2) how each topic is represented by specific descriptive terms and what terms are similar, and (3) what semantic relationships exist between resilience indicators. The resilience dictionary can improve the understanding of the semantic coverage of the meaning and realization of each resilience indicator. The dictionary comprises the features described in Textbox 1.

**Features of the resilience dictionary.**

**Resilience dictionary features**

- *Resilience indicator* indicates a resilience indicator.
- *Parent* indicates the direct parent resilience indicator for a given indicator identified in the resilience taxonomy.
- *Topic* provides a list of topics relevant to each resilience indicator determined using human knowledge.
- *Topic weight* indicates the average weight of each topic over time. To calculate this weight, we first found the topic most relevant to each post by identifying the highest membership weight of the post over topics from the document-ensemble topic matrix D. In a sense, this weight is also seen as the weight of the topic (the most significant topic) in the post. Second, we calculated the average weight of the most significant topics across all the posts. Thus, this feature represents the relative importance of each topic over all the topics.
- *Topic word* denotes the top 10 most descriptive terms of each topic.
- *Similar terms* characterize the synonyms of a topic term, aiming to increase the semantics of a topic term. As there is no existing synonym dictionary for web-based mental health communities, our approach exploits a machine learning technique called *word embedding*. Word embedding is used to identify synonyms of each topic term based on their co-occurrence with other terms in input forum posts. Note that word embedding tends to indicate similar words by identifying the nearest words that appear together in similar contexts. Thus, synonyms captured by word embedding may not be “pure” synonyms depending on the input context (eg, if 2 terms “good” and “bad” frequently co-occur together in similar contexts, these can be identified as synonyms). We used a model named word2vec [50] as a word-embedding model, which has demonstrated many advantages for analyzing the semantic analysis of words.

## Results

### Phase 1: Preprocessing of Mental Health Forum Posts

**Figure 4:**
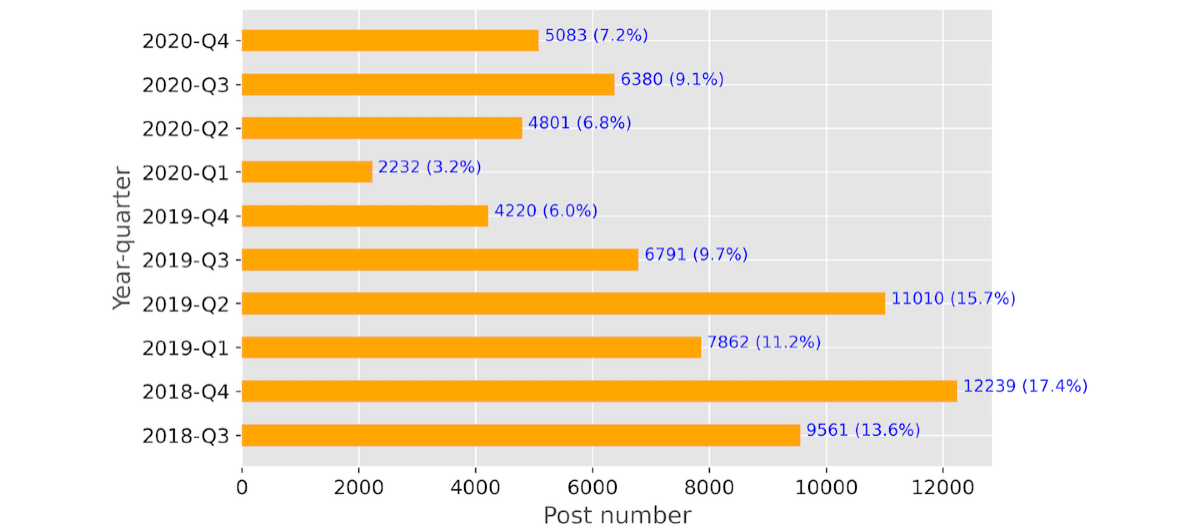
Summary of Schizophrenia: A National Emergency (SANE) forum posts in the sample 2018-Q3 to 2020-Q4.

From the original 70,179 posts posted by 2357 users, Figure 4 shows the number of posts by time quarter. As the input for topic modeling, we used 69.56% (48,819/70,179) of posts. Note that we removed 30.44% (21,360/70,179) of posts with a length of <5 words (ie, too short).

**Figure 5:**
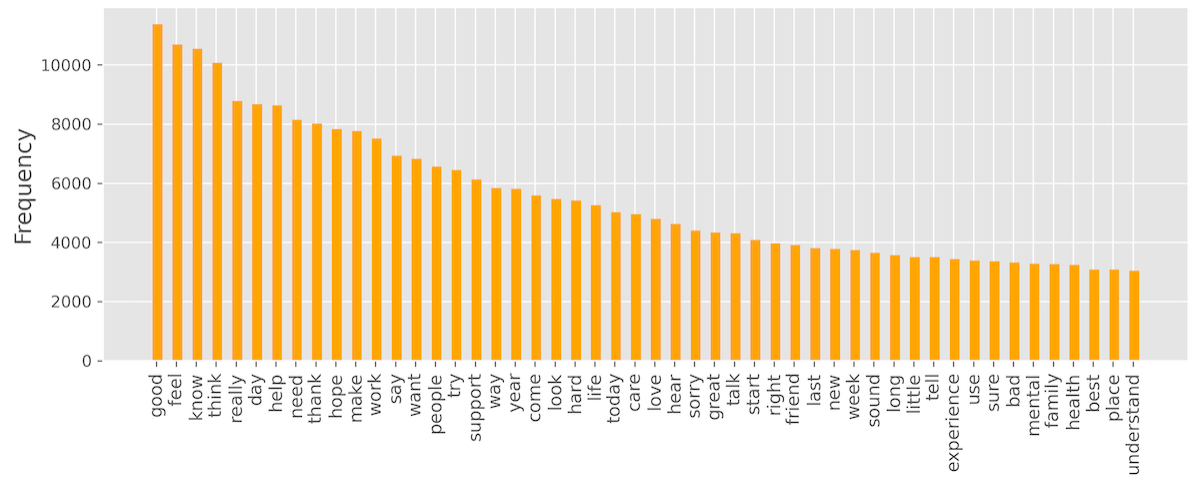
The top 50 terms by word frequency observed from the 48,819 posts.

Figure 5 shows the top 50 terms by word frequency in the post data. Most comprise a mixture of nouns (eg, *day*, *help*, *need*, *way*, and *year*), verbs (eg, *help*, *need*, *feel*, *know*, *think*, *want*, and *sorry*), and adjectives (eg, *good*, *hard*, *great*, and *little*), whereas adverbs were relatively less used (eg, *really*). In total, from the 48,819 posts, we extracted 14,938 terms after the preprocessing phase. The minimum, average, and maximum term counts of the posts were 6, 30, and 1182, respectively.

### Phase 2: Topic Modeling

**Figure 6:**
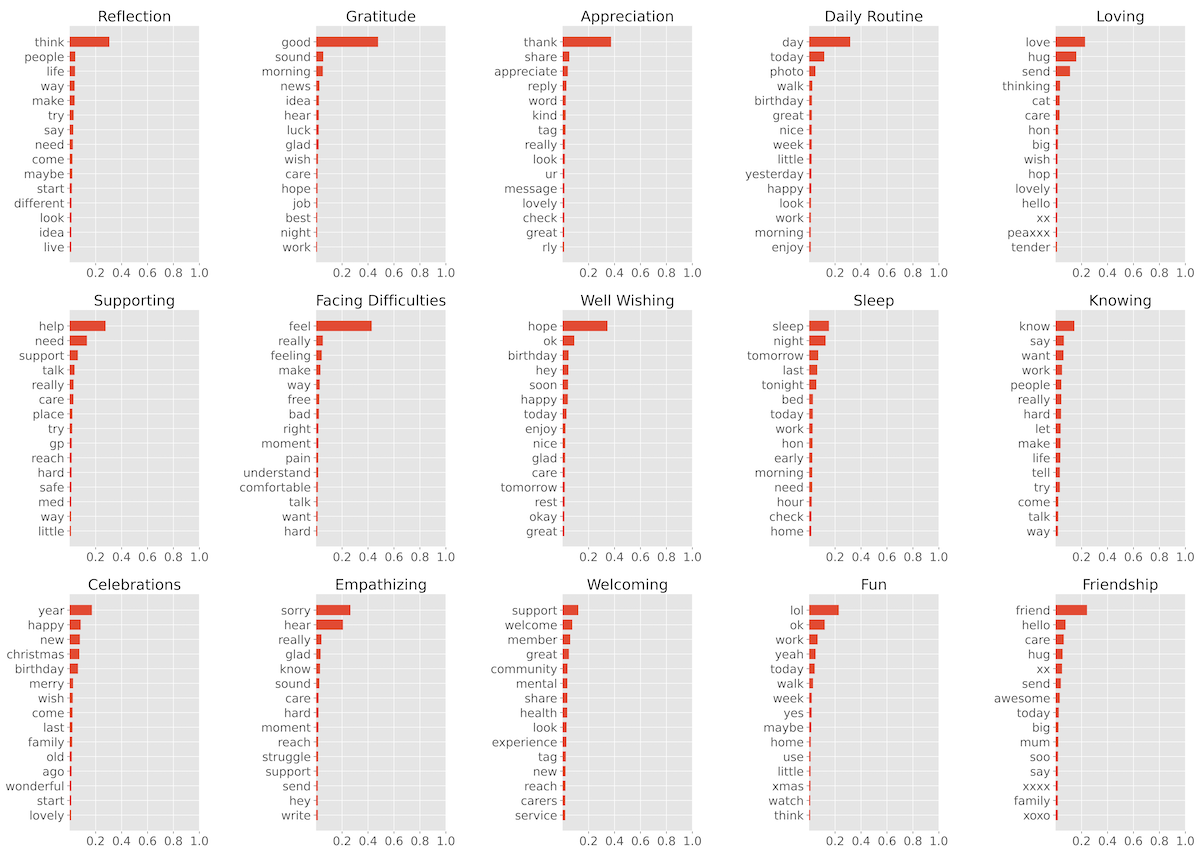
The 15 generated ensemble topics, where each topic is represented by its top 15 descriptive terms.

The 48,819 posts were split into 10 disjoint post collections based on quarters 2018 Q3 to 2020 Q4. From each collection, we discovered topics using NMF. To generate topics, *k* and *k′* must be provided, where *k* and *k′* determine the number of base and ensemble topics to be generated, respectively. There is no universal method of determining such numbers [43]. In our study, we used a fixed value of 10 as *k*, assuming that we equally discovered the 10 main topics being discussed from each collection. Initial experiments found *that k*=10 (range was *k*=5-15) produced an informative topic set that was neither too general nor too specific. To discover the *k′* ensemble topics, a more complex method was applied by varying *k′* from 10 to 20 in increments of 1 to choose an optimal number. As we did not know how many base topics were similar or different, we measured similarities and variances of the base topics over the entire timeline using a widely used metric, *topic coherence* [51]. This method measures the degree of similarity between the top *N* terms for each topic. From the perspective of topic coherence, a better-interpretable topic yields a higher average score for the topic coherence scores of all generated topics. To measure topic coherence, we used word embedding [43] to measure the similarity between the top *N* terms in a topic. This similarity was estimated using the cosine similarity between word-embedded vectors. For the value of *N*, we used 10, following the suggestion in the study by O’Callaghan et al [43]. We generated a word-embedding model from the entire post data using word2vec [50]. Finally, using topic coherence, we selected 15 as the optimal value for *k′*. Figure 6 shows the 15 ensemble topics generated. For each topic, the top 15 descriptive terms were ranked vertically (the first row is the most descriptive term) based on their weights (importance). The weight of each term in a topic is visualized by a “red bar,” where the sum of the weights of all terms in a topic is normalized to 1.

### Phase 3: Resilience Conceptualization

#### Results of Mapping Topics to Resilience Indicators

A qualitative thematic analysis following a previous work [52] was conducted by 4 researchers with expertise in qualitative mental health research (second, third, and fourth authors) to interpret the meaning of the topics and assign descriptive topic labels to them. These labels, as shown in Figure 6, describe each topic by finding the most suitable meaning conveyed by the top 15 words in the identified topics and the top 20 posts associated with that topic. First, the researchers familiarized themselves with the topic modeling results, independently noting the initial ideas that were used to provide a semantically representative label of topic words and posts. Subsequently, topic labels were compared and refined to achieve satisfactory interresearcher agreement. The final agreed topic labels were then attributed to ≥1 of the 5 resilience indicators by each researcher through a deductive process based on the established resilience theory. Finally, these groupings were reviewed by the team and refined for consistency (Table 2).

The identified topics reflect the forum’s aim to provide ongoing, accessible, peer-to-peer support for people experiencing mental health conditions. Although specific instances of mental ill health, such as depression and anxiety, were often discussed, they were not the focus of the identified topics; that is, although some topics expressed mental health problems directly, such as “sleep” and “facing difficulties,” daily challenges and indicators of social connection and support were more commonly identified topics. Topics also have an important temporal dimension, which is an advantage of our methodology, which considers topics over time. The 2-year sample of posts and topic modeling based on quarters meant that annual events or celebrations such as Christmas, New Year’s Eve, and birthdays were commonly referenced, showing the importance of social connection over time as a feature of the forum. Most topics expressed the establishment and maintenance of interpersonal social connection: “welcoming,” “empathising,” “well wishing,” “supporting,” “loving,” “friendship,” and “appreciation.” A few topics were more self-directed: “gratitude,” “reflection,” and “knowing.” The latter is important but rarer indicators of adaptation and a sense of self that is necessary for resilience.

The researchers attributed topics to resilience indicators deductively, considering each topic’s top 15 words and associated top 20 posts. No assumptions were made regarding whether topics fit all resilience indicators and whether topics could be attributed to 1, >1, or no indicator. Strong agreement was achieved among the researchers, with some minor differences noted and discussed before an agreement was reached. The results of mapping topics to resilience indicators are shown in Table 2, with example posts.

**Table 2:** Result of mapping topics to resilience indicators.

Resilience indicator	Mapped topic	Example posts
Social capital	Gratitude, appreciation, daily routine, loving, supporting, well wishing, empathizing, fun, and friendship	“[...] you did good???? so you did do the dishes eventually. Just organised my bedside table and tidied backyard (courtyard), will get back to it in the morn, ta” [Gratitude] “Woooohhhhooo thank you incredible human beings! Echoing x1000 [x’s] sentiment here” [Appreciation] “The way to get help out of those cycles is to talk about them. Would it help to write them in a piece of paper and hand that over to the pdoc?” [Supporting]
Belonging	Gratitude, appreciation, daily routine, loving, well wishing, celebrations, empathizing, welcoming, fun, and friendship	“Another welcome to the forum [...], it sounds like you’ve been through a lot so it’s understandable that you’re under so much stress.” [Welcoming] “Happy New Year everyone in NSW, Vic, ACT and Tassie” [Celebrations] “I get tired of my physical problems too but one step at a time my awesome friend, walking with you I am here for you all the way and so is [...]” [Friendship]
Learning	Reflection, supporting, and knowing	“Listening to music does help. Need to learn to dismiss those unhelpful negative voices.” [Supporting] “I’m trying so hard to see through my tears. it’s hard but I am determined to get there. Just knowing that you are here gives me strength x????” [Knowing]
Adaptive capacity	Reflection	“Will be thinking of you through this process [...] and pray that the transition is as smooth as possible.” [Reflection]
Self-efficacy	Facing difficulties and sleep	“Hi, I’m feeling very overwhelmed with anxiety atm. I’m trying to control it, but it feels like I’m a big shaking mess inside. I feel short of breath and my heart is racing” [Facing difficulties] “Anxiety is off the charts. I’m sleep deprived as I got zero sleep last night and my mood is extremely low.” [Sleep]

Most topics were attributed to the indicators “Social Capital” and “Belonging.” This aligns with the well-understood attributes of social media, which encourage and function through phatic communication, more so than informational or dialogic intents [53]. Within this broad sociopragmatic function of posts, we could see a range of topics that distinguished different methods of establishing and maintaining social connections. Topics associated with “Learning,” “Adaptive Capacity,” and “Self-efficacy” were rare, and these can be considered more explicit expressions of exhibited resilience, where change, adaptation, and coping become possible.

#### Results of Resilience Prevalence Analysis

**Figure 7:**
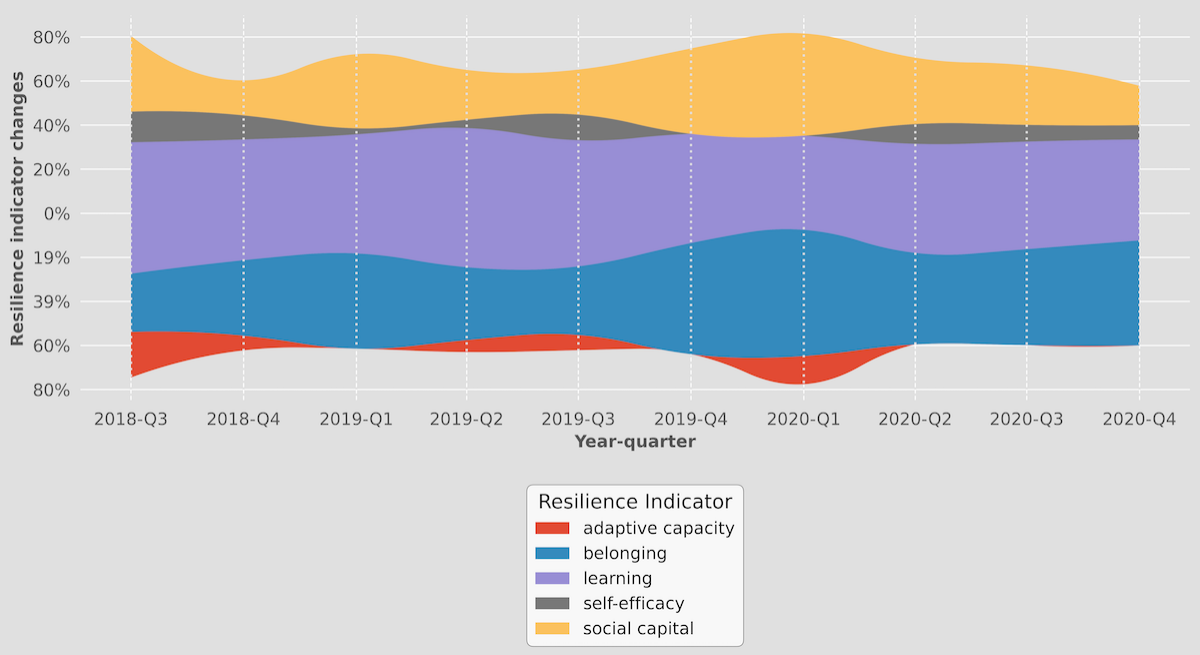
Resilience indicattor realizaion in Schizophrenia: A National Emergency (SANE) Australia.

Figure 7 shows the resilience streamgraph that resulted from the resilience prevalence analysis, which identified the dynamics of the realization of resilience indicators over time. This streamgraph was created to visualize our understanding of resilience prevalence [54], which is a popular method for displaying changes in different categories (ie, resilience indicators) over time. Instead of visualizing values as a conventional y-axis, streamgraphs offset the baseline of each “stack” to make it symmetrical around the x-axis. This results in a stream shape that illustrates the change in values over time. Through this streamgraph, we analyzed the patterns of resilience indicators in terms of their realization over different quarters represented by the x-axis.

A total of 3 findings can be summarized in Figure 7. First, the prevalence of each indicator changes according to the proportion of posts (y-axis), with the height of each individual stream shape showing the prevalence of each indicator over time. The y-axis is not positive or negative but rather shows the best stacking arrangement. Second, “learning” and “belonging” were the top 2 most realized indicators over the entire timeline. The next most common indicator was “social capital,” whereas “self-efficacy” and “adaptive capacity” were less realized during the timeline, with shallow stream shapes appearing and disappearing at different points in time. Third, there were no distinct seasonal peaks and periodic patterns for the top 3 dominant indicators (ie, “learning,” “belonging,” and “social capital”) over the timeline. However, both “belonging” and “social capital” were observed to be slightly stronger at 2020-Q1. By contrast, the realization of both “self-efficacy” and “adaptive capacity” noticeably changed over time: (1) “self-efficacy” was hardly realized at the periods 2019-Q1, 2019-Q2, 2019-Q4, and 2020-Q1, and (2) “adaptive capacity” also seemed to be very weakly realized at the periods 2019-Q1, 2019-Q4, and 2020-Q2 to 2020-Q4. Investigating the possible reasons for these observations may be a useful area for future research.

### Phase 4: Resilience Dictionary Building

#### Result of the Resilience Taxonomy Inference

**Figure 8:**
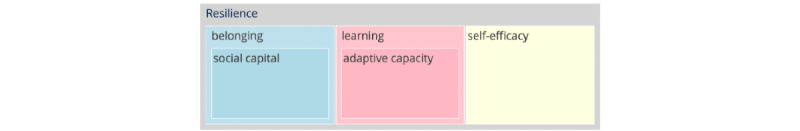
The resilience taxonomy induced from the SANE (Schizophrenia: A National Emergency) Australia posts represented as a treemap that shows the coverage of the concept of each resilience indicator by its rectangle size.

Given the co-occurrences of the resilience indicators mapped to the posts, we constructed a resilience taxonomy. We used a treemap representation (Figure 8) that shows the corealization of resilience indicators according to 2 levels. The first level has the indicators “belonging” and “learning.” These are higher-level and more general indicators. This means that these indicators were realized at a high prevalence, serving as foundational aspects of resilience. The indicator “social capital” is the more specific indicator subsumed by “belonging.” This means that whenever “social capital” is realized, “belonging” is most likely to be realized as well. The same association was applied to “learning” and “adaptive capacity.” From another perspective, we can also draw an interpretation relating to the coverage of the 5 resilience indicators, where the coverage of each indicator is indicated by rectangular sizes. “Social capital” is clearly realized in relation to “belonging,” and “adaptive capacity” is realized in relation to “learning.” Colors are used to indicate similar characteristics, where more specific indicators are represented by darker intensities of the same colors. The indicator “self-efficacy” was realized independently, regardless of the other 4 indicators. The emergent taxonomy can be used to gain insights into which indicators are more likely to corealize and what types of conceptual relations are observable between indicators, as inferred by their corealization.

#### Result of Resilience Dictionary Generation

Table 3 shows the created resilience dictionary, demonstrating (1) the parent of each resilience indicator represented by “Parent,” (2) topics relevant to each indicator obtained from Table 1 by “Topic,” (3) the relative importance of topics by “Topic Weight,” and 4) the top 10 descriptive terms of each topic by “Topic Words.” The 5 most similar terms for each descriptive term are presented in Multimedia Appendix 1.

In summary, 2 sets of insights were provided by the resilience dictionary. First, sets of topics associated with different resilience indicators were identified across the entire study period and within each quarter, revealing both constant and periodic topics. These topics and topic words form a useful set of semantic collections that can help characterize each resilience indicator. Second, topic weights, prevalence, and co-occurrences of topics associated with resilience indicators can be used to construct a resilience taxonomy. This taxonomy revealed foundational indicators of resilience—belonging and learning—and more specific and less prevalent indicators—social capital (subsumed by belonging) and adaptive capacity (subsumed by learning)–with self-efficacy realized independently of other indicators.

**Table 3:** The resilience dictionary constructed from this study.

Resilience indicator, parent, and topic				Topic weight (%)		Topic words
**Social capital**						
	**Belonging**					
		Gratitude	3.6		good sound morning news idea hear luck glad wish care	
		Appreciation	6.1		thank share appreciate reply word kind tag really look ur	
		Daily routine	7.7		day today photo walk birthday great nice week little yesterday	
		Loving	3.3		love hug send thinking cat care hon big wish hop	
		Supporting	2.4		help need support talk really care place try gp reach	
		Well wishing	1.6		hope ok birthday hey soon happy today enjoy nice glad	
		Empathizing	1.3		sorry hear really glad know sound care hard moment reach	
		Fun	1.6		lol ok work yeah today walk week yes maybe home	
		Friendship	1		friend hello care hug xx send awesome today big mum	
**Belonging**						
	Gratitude		3.6		good sound morning news idea hear luck glad wish care	
	Appreciation		6.1		thank share appreciate reply word kind tag really look ur	
	Daily routine		7.7		day today photo walk birthday great nice week little yesterday	
	Loving		3.3		love hug send thinking cat care hon big wish hop	
	Well wishing		1.6		hope ok birthday hey soon happy today enjoy nice glad	
	Celebrations		3.1		year happy new christmas birthday merry wish come last family	
	Empathizing		1.3		sorry hear really glad know sound care hard moment reach	
	Welcoming		11.5		support welcome member great community mental share health look experience	
	Fun		1.6		lol ok work yeah today walk week yes maybe home	
	Friendship		1		friend hello care hug xx send awesome today big mum	
**Learning**						
	Reflection		5.2		think people life way make try say need come maybe	
	Supporting		2.4		help need support talk really care place try gp reach	
	Knowing		44.8		know say want work people really hard let make life	
**Adaptive capacity**						
	**Learning**					
		Reflection	5.2		think people life way make try say need come maybe	
**Self-efficacy**						
	Facing difficulties		2.6		feel really feeling make way free bad right moment pain	
	Sleep		4		sleep night tomorrow last tonight bed today work hon early	

## Discussion

### Principal Findings

This paper presents a semiautomatic approach for generating a resilience dictionary from online mental health peer-support forum data. It applies a systematic 4-phase analysis pipeline (Figure 1) that preprocesses raw forum posts, discovers core themes, conceptualizes resilience indicators, and generates a resilience dictionary. We show the promise of exploring how resilience indicators are realized in a web-based mental health community to enrich the characterization of each resilience indicator by showing its range of topics or ways it is discussed and the interdependencies between different aspects of resilience.

A major contribution of this study is that it provides a replicable method for generating further resilience and other topics or theory-focused dictionaries. The proposed method pipeline can be applied to online support forums hosted by other mental health organizations to better understand their web-based communities and build a deeper understanding of the realization of resilience.

To address RQ1, we present a method for using both the 2-layered NMF topic modeling technique and human knowledge through thematic analysis to discover and label the 15 substantive topics and map them to the 5 resilience indicators. Each resilience indicator was characterized by its relevant topics (Tables 2 and 3). The analysis showed that we discovered topics related to establishing and maintaining social connections and bonding, building trust, and belonging over time, including through the sharing of milestone events such as Christmas, New Year’s Eve, and birthdays, as well as more routine moments. Reflection and knowledge sharing illustrate the importance of learning. In addition, sharing difficulties and ongoing issues associated with mental ill health such as lack of sleep illustrates the role of the forums for participants in navigating self-efficacy and agency amidst the mental health difficulties they face in their daily lives.

We traced the proportion of relevant posts over time to reveal the prevalence of the different aspects of resilience (RQ2). Figure 7 shows the dominance of “learning,” “belonging,” and “social capital,” with “self-efficacy” and “adaptive capacity” appearing less frequently. These findings also help answer RQ3, addressing the relationship between different resilience indicators. To further clarify, we developed a resilience dictionary that shows how each resilience indicator is characterized through a set of relevant topics, the most relevant descriptive terms (including their synonyms) for each topic, and the semantic relationships between resilience indicators observed from the input post data (Table 3 and Multimedia Appendix 1). A taxonomy was developed to establish the relationships of interdependence between different resilience indicators (Figure 8), emphasizing the relationship between generators (belonging and learning) and outcome-oriented (social capital, adaptive capacity, and self-efficacy) indicators of resilience, in line with existing resilience research [27].

A growing body of research has sought to assess the impact of web-based mental health peer support. Peer-support forums offer a form of health intervention that can provide support at scale; however, they also present challenges regarding their design to optimize benefits and their evaluation. New computational methods for NLP, combined with qualitative analysis, provide opportunities to address these challenges. We leveraged NLP-based statistical analysis and qualitative analyses to generate new insights. This work extends beyond the capacity of qualitative content analysis studies that have attempted to identify the effects on resilience through surveys or interviews, cross-sectionally and at a small scale [9,14,18,19,21].

Our work identifies strengths-based indicators of resilience and the relationships between them. This represents an advance or change over previous studies that tended to focus on risk and negative issues regarding mental health, including identifying negative symptoms [31,32], emotions [33,34], and potential risks of self-harm [23]. Although responding to risk and intervening to support people is important, so is the capacity to look at the strengths and resources generated through web-based forums. Complementing these approaches, our work takes a strengths-based approach, with the resultant resilience dictionary able to add a positive context to deepen the understanding of the forum impact and its most effective attributes.

The benefits of the resilience dictionary are that it can be used for (1) showing that resilience, as understood by the current theory, is realized through activity on forums; (2) improving understanding of the aspects of resilience realized on web-based forums; (3) building evidence-based resources for training staff and volunteers by showing how the different aspects of resilience are characterized and how resilience topics are embedded in certain discussions; (4) improving the design and automated moderation of mental health forums by enabling analysis to move beyond risk and harm to strengths-based indicators of resilience; and (5) aiding further text analysis as a vocabulary set for building resilience detection systems that can help mental health care providers to design to improve the outcomes of their services and illustrate their impact.

This study is significant for research and practice in potentially far-reaching areas. First, resilience itself, how it forms, and through what processes and means is poorly understood. Resilience tends to be an ill-defined concept in mental health practice. Here, we have shown how indicators, as part of a conceptual frame, resonate with activity and discussion over time in a web-based forum. Thus, by applying contemporary data analysis techniques to novel data, we have contributed to social and psychological knowledge about this contested concept. Second, for practice, this work shows the potential of (re)using novel data generated through a service to provide useful information that can help design and maintain those services, respond better to health consumers, and show their impact. These are all important issues for nonprofit organizations that struggle to secure and allocate scarce resources. This work should prompt mental health services to gear up their capacity to work with data as it shows considerable potential and innovative uses for data and data skills.

### Limitations

This study has some limitations that could be addressed in future research. First, it focused on creating a resilience dictionary from a single forum and data source (ie, SANE Australia). It would be useful to build resilience dictionaries from multiple data sources and explore similarities and differences. In doing so, we might gain insights into common and distinctive resilience indicators across forums. If resilience realization is different, this could be explored in relation to varying forums and organizational aims, management strategies, and user demographics. Second, the study is “static” in the sense that it used a large existing corpus of historical data. Considering that web-based mental health forums are a growing type of service, it could be useful to investigate how to enable a resilience dictionary to incrementally evolve in response to new forum data.

### Conclusions

In this paper, we presented a promising approach to creating a resilience dictionary that provides insights into the dominant topics on mental health peer-support forums and the way these topics realize different indicators of resilience. The developed mixed NLP and qualitative methods forge new grounds in helping forum providers analyze and understand the impact that the forums provide through a strengths-based analysis of resilience. Along with illustrating the prevalence of different resilience indicators over time, a taxonomy demonstrates the interdependence of different indicators, revealing which are foundational (belonging and learning) in relation to others (social capital and adaptive capacity). The resulting resilience dictionary offers a benchmark and vocabulary set that can aid further research. It can also be used to inform automated systems normally predicated on terms associated with risk, harm, and diagnostic indicators of mental ill health or distress, adding a strengths-based approach to the moderation of forum content and interactions.

## Appendix

Multimedia Appendix 1 Full resilience dictionary.

## Abbreviations

NLP: natural language processing
NMF: nonnegative matrix factorization
RQ: research question
SANE: Schizophrenia: A National Emergency
