# Supplementary material for: Resilience in Web-Based Mental Health Communities: Building a Resilience Dictionary With Semiautomatic Text Analysis
Source: JMIR Form Res. 2022 Sep 22;6(9):e39013. doi: 10.2196/39013 (PMC9539645; doi:10.2196/39013)
Supplement: Multimedia Appendix 1 [file formative_v6i9e39013_app1.pdf]

Multimedia Appendix: Resilience Dictionary [1]

No	Term	Nearest Neighbouring Terms	No	Term	Nearest Neighbouring Terms
1	ago	nearly, last, roughly, boozy, twice	67	mum	dad, aunty, sister, mil, cousin
2	appreciate	appreciated, thank, encouragement, thoughtful, encouraging	68	need	likewise, overstep, eventuate, hel, needs
3	awesome	fantastic, wonderful, congrats, fabulous, forumite	69	new	reconnecting, excited, thrill, forumi, trialling
4	bad	crappy, horrendous, horrible, worst, defeat	70	news	ta, luck, pleased, thsts, gday
5	bed	doze, bedtime, couch, nap, doona	71	nice	lovely, brighten, refreshing, relaxing, greet
6	best	sensible, kindest, speedy, maz, continued	72	night	slept, awake, sleeper, sleepless, bedtime
7	big	huge, massive, cyber, oxoxox, incoming	73	ok	okish, everythings, alright, alls, sheesh
8	birthday	belated, 50th, keeper, merry, youhappy	74	okay	alright, tabs, apprehensive, okish, alls
9	care	tske, pls, xxxooo, considerate, xxoo	75	old	older, younger, kindy, teenage, neice
10	carers	carer, le, lived, gateway, red-centaur	76	pain	killer, ache, painful, neck, spasm
11	cat	dog, toby, tabby, pet, companion	77	peaxxx	peaxx, sweety, ohhhhh, hunny, holly
12	check	check-in, inbox, fyi, checking, sent	78	people	understands, introvert, uncaring, alienate, likeminded
13	christmas	merry, xmas, nye, eve, easter	79	photo	picture, sunset, photograph, lookout, pic
14	come	agsin, unexpectedly, helpand, notch, ny	80	place	retreat, apprehension, eager, safer, nd
15	comfortable	intrude, apprehension, connected, safer, merrier	81	reach	posting, courageous, scbs, helplines, eden
16	community	collaborate, builder, cgs, guide, non-judgemental	82	really	definently, yiu, mustve, eloquent, realy
17	day	slump, youo, tomorrows, brissie, moving	83	reply	typo, msg, delayed, individually, replying
18	different	particular, certain, depend, vary, angle	84	rest	peacefully, recharge, restful, latley, well-deserved
19	early	mid, groggy, midday, riser, doze	85	right	wether, anyones, no-ones, yiu, macca
20	enjoy	enjoyable, brighten, joyful, enjoyed, relaxing	86	rly	bn, hav, wld, ne, hapn
21	experience	experienced, profound, widely, disconnection, ex-psychiatrist	87	safe	unsafe, incase, safer, safety, alex
22	family	confide, legged, aunt, relative, estrange	88	say	txt, indi, alway, apologize, thati
23	feel	felt, confused, infact, wallow, terrifying	89	send	sending, sent, incoming, xox, hugs
24	feeling	emotion, helplessness, diminish, hopelessness, feel	90	service	hotline, callback, weekday, chatsuicide, 10am-10pm
25	free	hesitate, comfortable, anonymously, noel, welcomeplease	91	share	sharing, joining, validating, welcoming, suited
26	friend	reconnect, xxxxx, xoxohello, reconnecting, xoxoxo	92	sleep	sleeping, sleepless, sleeper, awake, bearable
27	glad	pleased, hope, thsts, babe, youhope	93	soo	sooo, xxi, ohh, xxhello, xxxxx
28	good	gday, crappy, motivated, settled, tomorrows	94	soon	tomorrow, goodluck, meantime, shortly, goodnight
29	gp	psychologist, doctor, referral, gps, psychiatrist	95	sorry	mustve, pressured, reassuring, condolence, aw
30	great	fantastic, excellent, brilliant, wonderful, congrats	96	sound	glad, amazingly, mustve, itd, hear
31	happy	belated, forumite, 50th, youhappy, not-birthday	97	start	begin, restart, coworker, apprehensive, counciling
32	hard	difficult, diffucult, exhausting, fighting, harder	98	struggle	hinder, heaviness, depressing, tremendously, empathize
33	health	mental, mh, heath, allied, menatl	99	support	stoke, assistance, invaluable, suport, gratefully
34	hear	welcomeim, hearing, unload, babe, eden	100	tag	dropdown, symbol, fh, tagging, recieve
35	hello	friendi, everyonei, gang, xxand, forumletting	101	talk	tslk, discuss, speak, hsve, understands
36	help	hel, preoccupy, pschiatrist, practicing, mediation	102	tell	piss, say, txt, brian, councilor
37	hey	gday, mustve, froggy, heya, hamsolo	103	tender	ohhh, sending, ohhhh, ohhhhh, incoming
38	home	motel, packing, hossie, pickup, visitor	104	thank	thankyou, appreciate, heartfelt, thx, bryana

39	hon	sucky, presently, tht, hugs, babe	105	think	ar, infact, stride, seeing, off-line
40	hop	hope, hopefully, hoping, apprehensive, continued	106	thinking	xxxooo, hugs, xox, youperi, youtake
41	hope	hop, hoping, hopefully, glad, youhope	107	today	yesterday, tomorrow, afternoon, tomorrows, arvo
42	hour	hr, minute, daytime, midday, midnight	108	tomorrow	arvo, goodluck, afternoon, tomorow, today
43	hug	sending, cyber, xox, hugs, tender	109	tonight	goodnight, evening, chatting, nite, sleepy
44	idea	seeing, starter, icebreaker, brainstorm, summarise	110	try	myslef, somedays, boundries, helpfull, rut
45	job	casual, employment, interview, referee, unemployment	111	understand	empathise, misunderstand, invalidate, tremendously, reluctance
46	kind	encouraging, reaffirm, reciprocate, appreciated, considerate	112	ur	urself, hav, bn, rly, ne
47	know	alas, wether, pressured, no-ones, whinge	113	use	custom, e-moods, sudoku, handy, apps
48	last	ago, earlier, february, quarter, row	114	walk	walking, workout, park, hill, stroll
49	let	reassure, intrude, tha, letting, wether	115	want	whinge, everytime, intrude, know, noel
50	life	independently, closure, lifes, strive, cling	116	watch	tv, movie, netflix, footy, abc
51	little	soooooo, amygdala, oooh, snuggle, toby	117	way	ourself, rut, mindset, disillusion, bogged
52	live	living, knew, independently, reconnected, granny	118	week	month, wednesday, august, reschedule, wks
53	lol	um, yeah, soz, prob, umm	119	welcome	warmly, aboard, firstly, welcoming, forums
54	look	ff, regardshope, innovation, anticipation, milestone	120	wish	continued, xo, bryana, kindest, carehope
55	love	angel, lover, squishy, mumma, purrpurr	121	wonderful	fabulous, amazing, fantastic, awesome, forumite
56	lovely	nice, brighten, sweety, greet, shout-out	122	word	compliment, ramblings, heartfelt, reaffirm, sentence
57	luck	apt, thsts, tomorrows, goodluck, someday	123	work	searching, timeframe, working, trainer, step-children
58	make	making, rash, humor, infact, fo	124	write	writing, poem, journal, read, paragraph
59	maybe	fo, agenda, someday, probably, breather	125	xmas	easter, bday, nye, christmas, cruise
60	med	medication, anti, antipsychotic, sedative, antidepressant	126	xoxo	xxxxx, xoxohello, xox, xxhello, sending
61	member	cgs, contributor, memebers, cm, chime	127	xx	xox, xoxo, xoxohello, xxhello, bb
62	mental	health, menatl, allied, mh, clinically	128	xxxx	xoxohello, xxxxx, xoxo, xxhow, xxx
63	merry	belated, chrissy, forumite, 50th, halloween	129	yeah	um, yeh, prob, soz, yest
64	message	explaining, txt, messenger, msg, reply	130	year	retire, decade, month, mths, boozy
65	moment	somedays, sporadic, sucky, exhausting, anticipation	131	yes	aw, eloquent, waffle, definently, ditto
66	morning	afternoon, today, midday, phonecall, yesterday	132	yesterday	today, morning, midday, phew, afternoon

[1] This is a Multimedia Appendix to a full manuscript published in the J Med Internet Res. For full copyright and citation information see http://dx.doi.org/10.2196/jmir.39013”
